# Tissue culture-induced genetic and epigenetic variation in triticale (× *Triticosecale* spp. Wittmack ex A. Camus 1927) regenerants

**DOI:** 10.1007/s11103-015-0368-0

**Published:** 2015-09-03

**Authors:** Joanna Machczyńska, Janusz Zimny, Piotr Tomasz Bednarek

**Affiliations:** Department of Plant Physiology and Biochemistry, Plant Breeding and Acclimatization Institute-National Research Institute, 05-870 Błonie, Radzików, Poland; Department of Plant Biotechnology and Cytogenetics, Plant Breeding and Acclimatization Institute-National Research Institute, 05-870 Błonie, Radzików, Poland

**Keywords:** Androgenesis, Demethylation, De novo methylation, Doubled haploid, In vitro culture, Sequence variation, Somatic embryogenesis

## Abstract

Plant regeneration via in vitro culture can induce genetic and epigenetic variation; however, the extent of such changes in triticale is not yet understood. In the present study, metAFLP, a variation of methylation-sensitive amplified fragment length polymorphism analysis, was used to investigate tissue culture-induced variation in triticale regenerants derived from four distinct genotypes using androgenesis and somatic embryogenesis. The metAFLP technique enabled identification of both sequence and DNA methylation pattern changes in a single experiment. Moreover, it was possible to quantify subtle effects such as sequence variation, demethylation, and de novo methylation, which affected 19, 5.5, 4.5 % of sites, respectively. Comparison of variation in different genotypes and with different in vitro regeneration approaches demonstrated that both the culture technique and genetic background of donor plants affected tissue culture-induced variation. The results showed that the metAFLP approach could be used for quantification of tissue culture-induced variation and provided direct evidence that in vitro plant regeneration could cause genetic and epigenetic variation.

**Electronic supplementary material:**

The online version of this article (doi:10.1007/s11103-015-0368-0) contains supplementary material, which is available to authorized users.

## Introduction

Triticale (× *Triticosecale* spp. Wittmack ex A. Camus 1927) is a fertile amphiploid derived from chromosome doubling of hybrids between two genera *Triticum* and *Secale*. Hexaploid forms have 42 chromosomes: 28 from wheat and 14 from rye (*Secale cereale* L.). The wheat complement of primary synthetic forms contained A and B genomes of durum wheat (*Triticum durum* Desf.) or other tetraploid species; in the contemporary cultivars, classified as the secondary triticales, majority of wheat chromosomes have been introgressed from hexaploid common wheat (*Triticum aestivum* L.). Triticale was artificially created with the aim of combining the productivity of wheat with the hardiness of rye (McGoverin et al. 2011). Triticale has excellent tolerance to water-limitation and salinity stress and displays high mineral efficiency (Blum 2014). Triticale is currently used for food, animal feed, and biofuel production (Hills et al. 2007), and is suitable for erosion control and as a cover crop (Ramirez-Garcia et al. 2015). The expansion of triticale cultivation has increased the need for improved breeding methodologies. One such technique is in vitro culture plant regeneration, which can provide breeders with homozygous lines (doubled haploids; DHs) in a single generation. DHs can be used for hybrid breeding, which is one of the most promising avenues for triticale improvement (Oettler et al. 2005). However, use of tissue culture can result in tissue culture-induced variation (TCIV) in regenerants (Dennis et al. 1987; Kaeppler and Phillips 1993a, b; Olhoft and Phillips 1994; Kaeppler et al. 1998) or somaclonal variation in progeny of the in vitro regenerated plants (Lorz and Scowcroft 1983; Larkin et al. 1984; Barwale and Widholm 1987; Breiman et al. 1987; Zehr et al. 1987; Bernardi et al. 1999; Kirikovich et al. 2003). Plant uniformity in in vitro regenerants and their progeny is compromised as a result of DNA methylation changes, cytological aberrations, transposon activation, and genomic variation (Phillips et al. 1994; Bednarek et al. 2007; Guo et al. 2007; Li et al. 2007; Ngezahayo et al. 2009; Baránek et al. 2010; Dann and Wilson 2011; Stroud et al. 2013; Wang et al. 2013; Zhang et al. 2014). Genetic and epigenetic changes induced at the DNA level due to plant tissue culture manipulation have been analyzed using a range of molecular markers (Polanco and MaL 2002; Xu et al. 2004; Smykal et al. 2007). More recently, the development and refinement of the metAFLP approach (Bednarek et al. 2007) allowed simultaneous quantification of sequence changes and DNA methylation patterns. The metAFLP method employs two isoschizomers, *Kpn*I and *Acc*65I, which differ in their sensitivity towards recognition site DNA methylation. *Kpn*I is insensitive and *Acc*65I is sensitive to restriction site methylation. This results in methylation-dependent differences in the amplified fragment length polymorphism (AFLP profiles) produced by the two enzymes, whereas sequence variation could be revealed based on the *Kpn*I/*Mse*I platform. AFLP profiles produced by metAFLP can be used for the identification of TCIV events and calculation of their quantitative characteristics. This technique has been used in a number of species to date, such as *Hordeum vulgare* (Bednarek et al. 2007), *Gentiana pannonica* (Fiuk et al. 2010), *Phyllostachys praecox* (Lu et al. 2012), and *Poa annua* (Chwedorzewska and Bednarek 2012). The ability of the extended metAFLP approach to characterize sequence changes as well as DNA methylation pattern alterations was demonstrated on limited triticale materials (Machczyńska et al. 2014a). Recently, global DNA methylation changes in triticale were analyzed using an RP-HPLC approach (Machczyńska et al. 2014b). This showed that global DNA methylation decreased in regenerants relative to the donor plants, but then increased in regenerant progeny. While RP-HPLC can be used for the analysis of global DNA methylation (Mankessi et al. 2011; Teyssier et al. 2013), this approach is not adequate for estimating subtle effects such as de novo methylation, demethylation, and sequence mutations that occur during in vitro plant differentiation and dedifferentiation (Zhang et al. 2010). It is not clear to what extent the metAFLP approach can identify the methylation changes identified by RP-HPLC or whether comparable estimates of similar characteristics, such as genome methylation (GM) and global DNA methylation, are derived via the two approaches independently.

The aim of this study was to use metAFLP to quantify TCIV in several triticale donor-regenerant sets developed using different androgenesis and somatic embryogenesis processes. In addition, metAFLP and RP-HPLC methods of GM and global DNA methylation were compared.

## Materials and methods

### Plant material

Four genotypes of DH regenerants derived from isolated microspores of triticale (Oleszczuk et al. 2004) were extracted from randomly chosen plants of the partly heterogeneous cv. Bogo (as it originated as a double cross) and were cloned by partitioning plant clumps after tillering. These served as explants for plant production via androgenesis in shed microspore culture (M) and anther culture (A), and by somatic embryogenesis from immature zygotic embryo culture (E) (Machczyńska et al. 2014b). Cloned individuals were kept in a growth chamber at a photoperiod of 16/8 h day/night at 16/12 °C to allow tillering. The same procedure (tillering and partitioning) was repeated every 2 weeks. Chosen cloned individuals (later called ‘donors’) of the four genotypes, their DH androgenic regenerants and homozygous regenerants derived from E, constituted the four sets (S^i^, where i = successive genotypes from 1 to 4) (Table 1).

**Table 1:** *Triticosecale* Wittmack cv. Bogo plant material used for metAFLP analysis

Set	Donors	Regenerants		
S^i^	D^i^C_n_	R_A_	R_M_	R_E_
S^1^	9	25	12	25
S^2^	8	20	8	15
S^3^	12	22	5	15
S^4^	10	12	12	17
Total	39	79	37	72

S^i^ represents four sets (i = 1–4); D^i^C_n_ indicates the number of donors used for in vitro plant regeneration, where D^i^ indicates donor DH genotype (i = 1–4) and C_n_ indicates cloned individuals; R_A_ represents regenerants derived from anther cultures; R_M_ represents regenerants derived from shed microspore cultures; and R_E_ represents regenerants derived from immature zygotic embryo cultures

### MetAFLP procedure

DNA was extracted from fresh leaves of donor plants and their regenerants at the same developmental stage (flag leaf emerging) using a DNeasy Plant Mini Kit (Qiagen). DNA samples were characterized spectrophotometrically following verification of integrity and purity on 1.2 % agarose gels with ethidium bromide staining.

MetAFLP was performed as described elsewhere (Bednarek et al. 2007). Following adapter ligation, pre-selection, and selective amplification steps, samples were digested with *Acc*65I/*Mse*I and *Kpn*I/*Mse*I endonucleases pairs (37 °C for 3 h, followed by 70 °C at 15 min). The arrangement of adapter and primer sequences is presented in Online Resource 1. PCR products were separated on a 7 % polyacrylamide gel. The metAFLP profiles for the *Acc*65I/*Mse*I and *Kpn*I/*Mse*I AFLP platforms were scored as ‘1’ (the presence of a band) and ‘0’ (absence), and arranged in a form of a binary juxtaposed matrix.

#### MetAFLP characteristics

Theoretically, sixteen four-digit binary codes were possible (Bednarek et al. 2007). The first and third positions of the binary code indicated the presence or absence of a marker in the AFLP profile of a donor plant (D) digested with *Acc*65I/*Mse*I and *Kpn*I/*Mse*I, respectively. The second and fourth positions reflected the same situation but for the regenerant (R). Four-digit binary codes were grouped into various events reflecting the different genetic background of those events. Genetic background sequence, demethylation, de novo methylation, and complex events were distinguished using the binary code and these reflected total tissue culture-induced events. Sites with non-methylated and methylated status in D and R were also identified. Sequence (SE), demethylation (DME), de novo methylation (DNME), and complex (CE) events were converted into sequence (SV), demethylation (DMV), de novo methylation (DNMV), and complex (CV) variation percentages using previously described formulae (Machczyńska et al. 2014a). Complex variation consisted of SV, DMV, and DNMV. These types of variation were extracted from CV and added to SV, DMV, and DNMV as a correction. All types of variation taken together described total tissue culture-induced variation (TTCIV). The metAFLP approach allowed quantification of non-methylated (SNMS) and methylated (SMS) sites. SNMS sites were those that remained non-methylated in donors (D) and regenerants (R), as well as those that underwent demethylation in regenerants. Sites that were methylated in D and R plus those that were *de novo* methylated in R were classified as SMS. Sites affected by methylation (SAM) were calculated as the sum of DNMV and DMV. Finally, metAFLP was used to evaluate the percentage of global genome restriction sites that were methylated in regenerants (GM). GM was defined as the sum of DNME and SMS divided by the sum of DNME, DME, SMS, and SNMS multiplied by 100. Detailed information regarding the quantitative characteristics of the metAFLP approach are published elsewhere (Machczyńska et al. 2014a).

### Global DNA methylation evaluation using RP-HPLC

Briefly, 6 µg of DNA from each sample was enzymatically hydrolyzed to nucleotides using P1 nuclease and then dephosphorylated with alkaline phosphatase. The reaction mixture was centrifuged at 12,000 rpm for 5 min and used for reversed-phase liquid chromatography (RP-HPLC) analysis (Machczyńska et al. 2014b). A Waters 625 LC Chromatography System connected to a Millennium 32v 4.0 data processing station was used for nucleoside separation. RP-HPLC analysis was based on the protocol described by Johnston et al. (2005). Two eluents were used (eluent A: 0.5 % methanol in 10 mMKH_2_PO_4_ (v/v), and eluent B: 10 % methanol in 10 mM KH_2_PO_4_) with a linear gradient of 10 min of 100 % A and 100 % B, and 15 min with 100 % B and 100 % A, with 5 min of total running time. The percentage of 5mdC (5-methyldeoxycytidine) was quantified as total 5mdC content divided by the sum of 5mdC and dC (deoxycytidine) multiplied by 100. Three analytical measurements were performed for each DNA sample.

### Data analysis

Mean and standard deviation (SD) values were calculated for metAFLP characteristics irrespective of sets or the in vitro tissue culture plant regeneration approach used. MetAFLP mean characteristics were also independently evaluated for all genotype sets and regenerants derived via anther culture, shed microspore culture, and immature zygotic embryo culture.

#### UPGMA

PAST software was used to analyze the metAFLP profiles of cloned individuals by UPGMA using Jaccard’s coefficient with 1000 bootstraps to estimate the robustness of the branches (Hammer et al. 2001).

#### ANOVA

R CRAN software was used for one-way ANOVA analysis with Tukey’s test to evaluate the differences between datasets. To avoid data correlation, sets were analyzed using uncorrelated metAFLP characteristics. The overall differences between the four sets as well as differences related to the in vitro tissue culture regeneration approaches and in vitro-induced variation characteristics were regarded as significant at a probability level of *p* ≤ 0.05 and α = 0.01.

#### Pearson correlation

Pearson correlation analysis was conducted for GM of metAFLP and global DNA methylation of RP-HPLC regenerant data (Machczyńska et al. 2014b) using the SAS statistical package (SAS Institute Inc 2004).

## Results

### Uniformity of doubled haploid cloned individuals

**Fig. 1:**
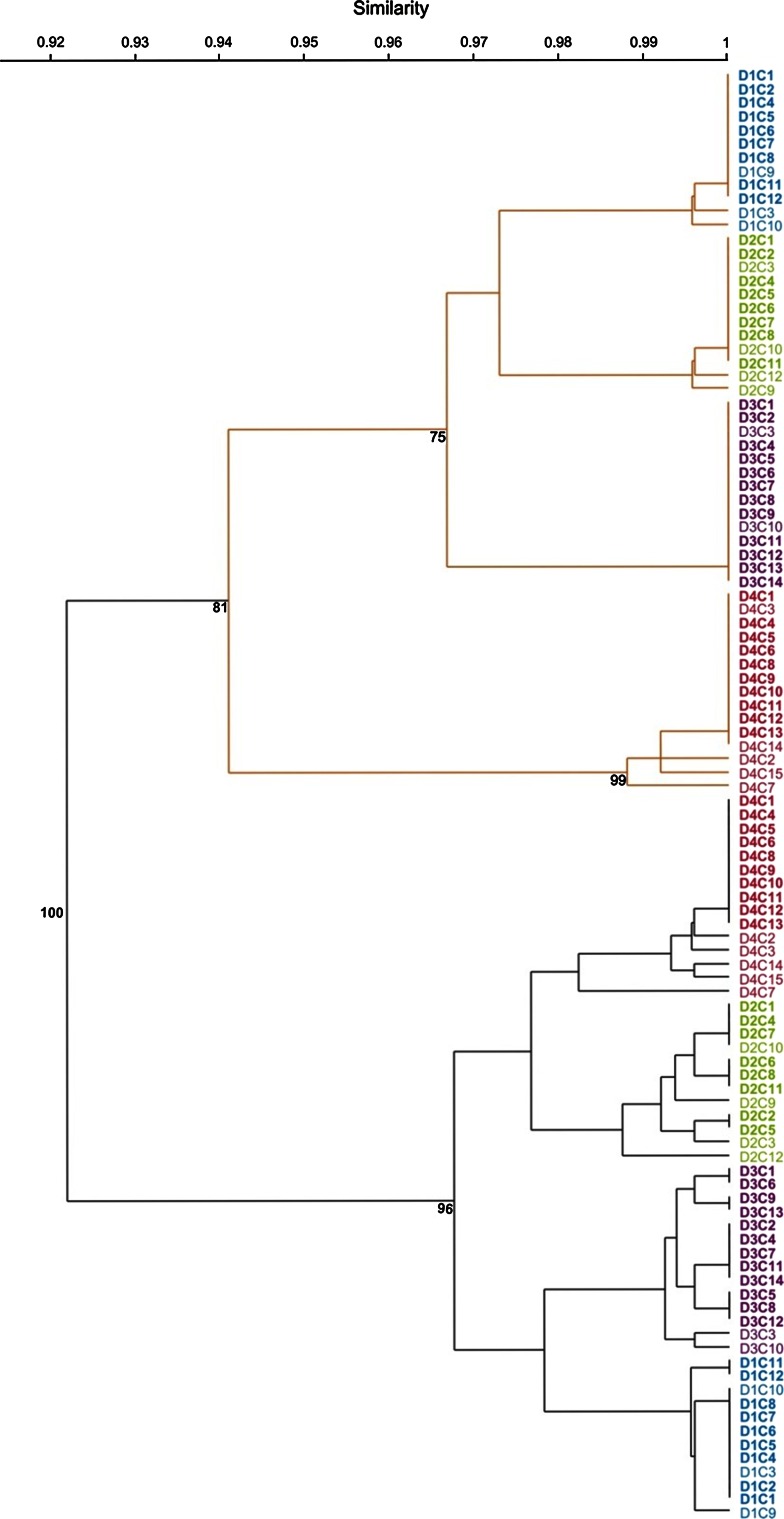
Clustering (UPGMA and Jaccard’s similarity index) of metAFLP data from 53 cloned individuals. Each individual is coded D^i^C_n_, where D^i^ represents donor DH genotype (i = 1–4) and C_n_ represents cloned individuals. The lower (*black*) cluster reflects *Acc65*I/*Mse*I data and the upper (*orange*) cluster reflects *Kpn*I/*Mse*I data. Cloned individuals of the same genotype are marked with the same *color*. Cloned individuals used for in vitro plant regeneration are shown in *bold*. Bootstrap values are indicated at the nodes

In total, 53 cloned individuals with no obvious morphological differences representing four distinct DH genotypes of cv. Bogo (D^i^C_n_, where D^i^ represents donor DH genotype (i = 1–4) and C_n_ represents cloned individuals) were obtained. Cluster analysis based on 2720 metAFLP markers amplified with 14 selective primer pairs distinguished two separate data groups that reflected the *Acc65*I/*Mse*I and *Kpn*I/*Mse*I digests (Fig. 1). Grouping based on *Acc65*I/*Mse*I-derived markers placed the D^1^C and D^3^C individuals in one group and D^2^C and D^4^C individuals in a second cluster. The *Kpn*I/*Mse*I metAFLP platform grouped D^1^C and D^2^C together, while D^3^C and D^4^C formed two subclusters with D^4^C being most distinct from the others. The genetic distances between individuals of the same genotype were 0.012 and 0.018 for the *Kpn*I/*Mse*I and *Acc65*I/*Mse*I platforms, respectively. In total, 9, 8, 12, and 10 genetically and epigenetically uniform cloned DH individuals (donors) representing four genotypes (D^1^, D^2^, D^3^, and D^4^) were produced and used for in vitro regeneration.

### Molecular characteristics of regenerants derived from uniform donor plants

Donor plants were a source of explant tissue for regeneration of 79, 37, and 72 plants via A, M, and E methods, respectively (Table 1). Spontaneously doubled androgenesis-derived regenerants and regenerants derived via immature zygotic embryos exhibited no apparent morphological differences compared to the donors.

Amplification using 14 selective primer combinations was performed on DNA samples from donors and their regenerants from all four genotype sets. In total, 2720 bands were produced with an average of 49 products per primer combination. The highest number of amplified fragments was observed for the CpXpG AGA/M CAA and the lowest was for the CpG GCA/M CGC primer combinations. In total, there were 1429 polymorphic and 1291 monomorphic fragments in *Acc65*I/*Mse*I, and 1172 and 1544 in *Kpn*I/*Mse*I, respectively (Online Resource 2). When all genotype sets were considered, a total of 1072 polymorphic and 935 monomorphic fragments were shared between the *Acc65*I/*Mse*I and *Kpn*I/*Mse*I digests.

### MetAFLP results

MetAFLP marker data were converted to four-digit binary codes. Codes (1111) related to non-methylated sites in donor and regenerant were most abundant (range 1865–12181, depending upon set and in vitro tissue culture regeneration method) (Online Resource 3). The less frequent events were those encoded as 0110 (range 22–95) and classified as demethylation and sequence events.

Calculation of the mean values of the metAFLP characteristics without separate consideration of the in vitro tissue culture regeneration methods or genotype sets used showed that 19.16, 5.48, 4.48, and 29.12 % of sites changed with respect to SV, DMV, DNMV, and TTCIV, respectively (Table 2). Up to 61.06 % of sites had non-methylated status in donors and regenerants (SNMS), and up to 4.33 % of sites were methylated (SMS). The percentage of SAM (10.59 %) was lower than the level of GM (12.19 %), as determined from methylation of the restriction sites and their vicinities.

**Table 2:** Mean quantitative metAFLP characteristics for different genotype sets and in vitro tissue culture regeneration methods

MetAFLP quantitative characteristics	S^i^	R_A_ (%)	R_M_ (%)	R_E_ (%)	Mean (all regeneration approaches) (%)
Sequence variation (SV)	S^1^	11.02 ± 0.93	11.62 ± 1.62	11.49 ± 0.62	11.38 ± 1.18
	S^2^	19.73 ± 1.14	18.55 ± 1	19.88 ± 0.75	19.64 ± 1.04
	S^3^	26.37 ± 0.76	25.76 ± 0.82	21.45 ± 0.82	24.54 ± 2.26
	S^4^	20.87 ± 0.9	20.67 ± 0.92	21.56 ± 0.74	21.10 ± 0.9
	Mean (all sets)	19.5 ± 5.51	19.15 ± 5.19	18.6 ± 4.35	19.16 ± 5.21
Demethylation (DMV)	S^1^	4.5 ± 0.54	4.07 ± 0.6	4.88 ± 0.32	4.48 ± 0.6
	S^2^	5.67 ± 0.75	6.28 ± 0.3	6.22 ± 0.57	5.94 ± 0.64
	S^3^	5.34 ± 0.36	5.35 ± 0.47	5.74 ± 0.36	5.48 ± 0.42
	S^4^	6.12 ± 0.43	5.86 ± 0.66	6.12 ± 0.49	6.04 ± 0.53
	Mean (all sets)	5.4 ± 0.75	5.39 ± 0.99	5.74 ± 0.7	5.48 ± 0.86
De novo methylation (DNMV)	S^1^	2.84 ± 1.14	3.58 ± 0.99	3.04 ± 0.79	3.18 ± 1.02
	S^2^	5.48 ± 1.61	4.08 ± 1.1	4.63 ± 0.85	5.0 ± 1.35
	S^3^	4.85 ± 1.02	5.06 ± 0.78	4.35 ± 0.61	4.71 ± 0.87
	S^4^	5.29 ± 0.74	5.21 ± 1.05	4.71 ± 0.75	5.03 ± 0.86
	Mean (all sets)	4.61 ± 1.59	4.48 ± 1.16	4.18 ± 1.02	4.48 ± 1.29
Total tissue culture-induced variation (TTCIV)	S^1^	18.36 ± 1.43	19.28 ± 1.8	19.41 ± 0.91	19.02 ± 1.48
	S^2^	30.88 ± 0.89	28.92 ± 0.9	30.73 ± 0.84	30.59 ± 1.1
	S^3^	36.55 ± 1.14	36.17 ± 0.83	31.55 ± 0.93	34.73 ± 2.36
	S^4^	32.28 ± 1.1	31.74 ± 1.25	32.39 ± 1	32.17 ± 1.11
	Mean (all sets)	29.51 ± 6.84	29.02 ± 6.46	28.52 ± 5.55	29.12 ± 6.5
Genome methylation (GM)	S^1^	7.95 ± 1.84	9.21 ± 1.34	7.07 ± 1.11	8.14 ± 1.74
	S^2^	16.33 ± 2.03	14.80 ± 1.5	15.17 ± 1.83	15.73 ± 1.83
	S^3^	10.71 ± 1.39	10.88 ± 0.86	10.54 ± 0.89	10.68 ± 1.13
	S^4^	14.45 ± 1.09	14.89 ± 1.43	13.63 ± 1.01	14.24 ± 1.24
	Mean (all sets)	12.36 ± 2.58	12.44 ± 1.96	11.6 ± 2.55	12.19 ± 2.4
Sites affected by methylation (SAM)	S^1^	7.34 ± 1.07	7.66 ± 1.18	7.92 ± 0.85	7.64 ± 1.06
	S^2^	13.66 ± 1.3	12.56 ± 0.8	13.52 ± 0.75	13.48 ± 1.08
	S^3^	10.19 ± 0.99	10.41 ± 0.78	10.09 ± 0.87	10.19 ± 0.96
	S^4^	11.41 ± 0.76	11.07 ± 0.99	10.83 ± 0.87	11.07 ± 0.91
	Mean (all sets)	10.65 ± 2.54	10.42 ± 2.27	10.59 ± 2.12	10.59 ± 2.36
Sites with methylated status in donor and regenerant (SMS)	S^1^	3.94 ± 0.68	4.29 ± 0.67	2.97 ± 0.32	3.76 ± 0.8
	S^2^	4.42 ± 0.75	4.60 ± 0.6	4.03 ± 0.43	4.23 ± 0.6
	S^3^	3.13 ± 0.42	3.13 ± 0.43	3.94 ± 0.49	3.42 ± 0.57
	S^4^	5.93 ± 0.45	6.26 ± 0.55	5.71 ± 0.4	5.94 ± 0.5
	Mean (all sets)	4.35 ± 1.03	4.57 ± 1.09	4.16 ± 1.02	4.33 ± 1.08
Sites with non-methylated status in donor and regenerant (SNMS)	S^1^	70.8 ± 1.05	69.71 ± 1.29	70.56 ± 0.85	70.32 ± 1.17
	S^2^	58.65 ± 1.6	60.40 ± 0.6	59.46 ± 1.01	59.15 ± 1.43
	S^3^	56.57 ± 0.92	56.92 ± 0.85	60.19 ± 0.53	57.91 ± 1.72
	S^4^	56.88 ± 0.86	56.78 ± 1.04	56.93 ± 0.73	56.87 ± 0.85
	Mean (all sets)	60.72 ± 5.89	60.95 ± 5.54	61.78 ± 5.37	61.06 ± 5.7

Percentages of sites affected are shown. R_A_, anther culture-derived regenerants; R_M_, shed microspore-derived regenerants; R_E_, immature zygotic embryo-derived regenerants. S^i^ (where i = 1–4) indicates genotype sets encompassing donor plants from four genotypes of DH of triticale cv. Bogo and their regenerants. Standard deviation is shown (±)

When regeneration methods (but not genotype sets) were considered, somatic embryogenesis-derived regeneration (R_E_) yielded the lowest values for SV, DNMV, and TTCIV as well as for GM and SMS. DMV and SAM values were lowest with the shed microspore method (R_M_). Values for all the quantitative characteristics were highest with anther-based regeneration (R_A_). When genotype sets were considered, values of SV, DMV, DNMV, TTCIV, GM, and SAM were generally lowest with the S^1^ set. Each set exhibited metAFLP values that varied with the different regeneration approaches (Table 2).

### ANOVA analysis

#### Comparison of sets by means of mean value of all metAFLP characteristics

**Fig. 2:**
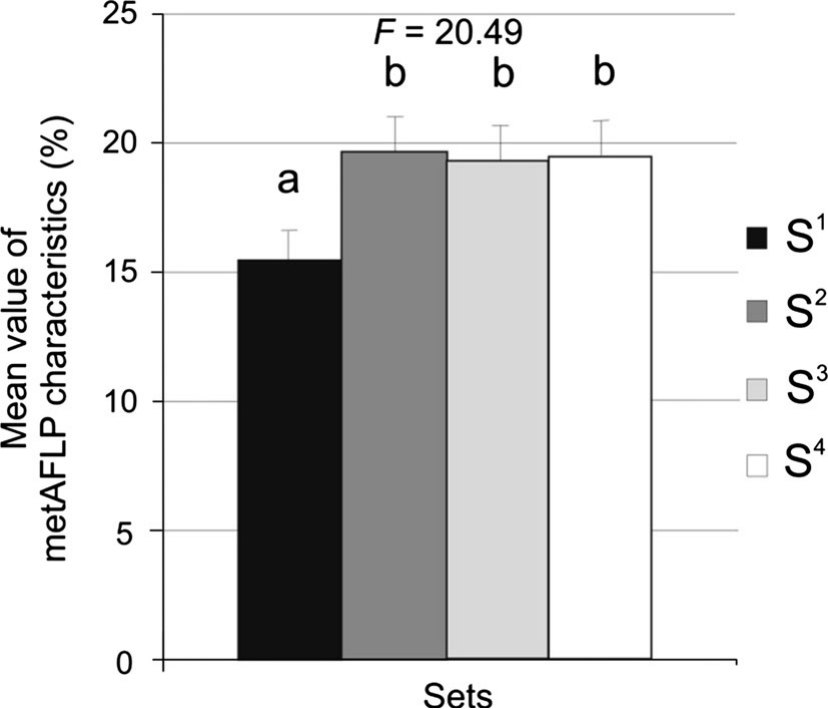
Grouping of genotype sets (Tukey’s multiple range test at 5 % significance level) using mean values of all metAFLP characteristics with *error bars*. Statistically similar sets (S^i^, where i = 1–4) are indicated with the same letter. *F* indicates ANOVA statistics (*p* ≤ 0.05 and α = 0.01)

Genotype sets were compared, independent of the tissue culture plant regeneration method used, by examination of mean metAFLP values (for *F* statistics see Fig. 2). Tukey analysis grouped S^2^, S^3^, and S^4^ together, but S^1^ was distinct (Fig. 2).

#### Comparison of sets by individual metAFLP characteristics

**Fig. 3:**
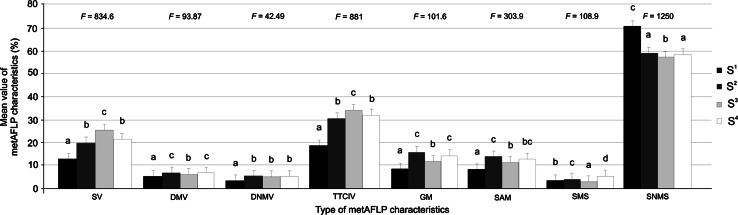
Grouping of genotype sets (Tukey’s multiple range test at 5 % significance level) for each metAFLP characteristic with *error bars*. SV, sequence variation; DMV, demethylation variation; DNMV, de novo methylation variation; TTCIV, total tissue culture-induced variation; GM, genome methylation; SAM, sites affected by methylation; SMS, sites with methylated status in donor and regenerants; SNMS, sites with non-methylated status in donor and regenerants. For each characteristic, statistically similar sets (S^i^, where i = 1–4) are indicated with the same letter. *F* indicates ANOVA statistics (*p* ≤ 0.05 and α = 0.01)

Most genotype sets differed with regard to their metAFLP characteristics (for *F* statistics see Fig. 3). Tukey analysis differentiated the sets into three broad groups: S^1^, S^3^, and S^2^ with S^4^. Specifically, S^2^ and S^4^ significantly differed only with respect to SMS and were not significantly different for SV, DMV, TTCIV, GM, and SNMS. For DNMV, S^2^, S^3^, and S^4^ formed a single group that was distinct from S^1^. For SAM, S^1^, S^2^, and S^3^ differed from each other, but S^4^ was similar to both S^2^ and S^3^ (Fig. 3).

#### Comparison of in vitro tissue culture regeneration methods

No statistically significant differences were observed between the different regeneration methods (R_A_, R_M_, and R_E_) when genotype sets and different metAFLP characteristics were considered together (ANOVA *F* = 0.145).

#### Comparison of genotype sets and different plant regeneration methods with overall metAFLP values

**Fig. 4:**
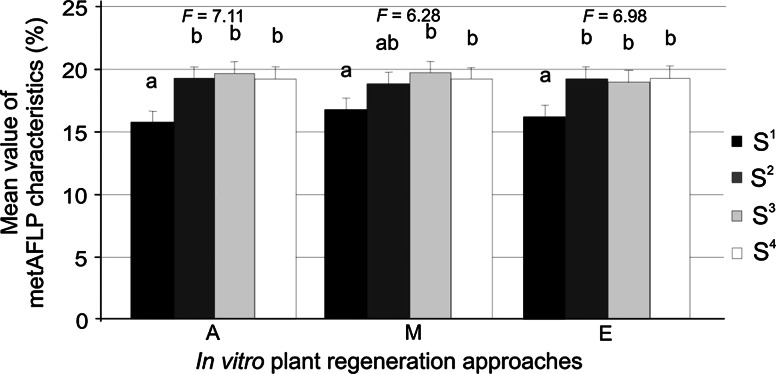
Grouping of genotype sets (Tukey’s multiple range test at 5 % significance level) for mean metAFLP characteristics with respect to plant regeneration method with *error bars*. A, anther culture-derived regeneration; M, shed microspore-derived regeneration; E, immature zygotic embryo-derived regeneration. For each regeneration approach, statistically similar genotype sets (S^i^, where i = 1–4) are indicated with the same letter. *F* indicates ANOVA statistics (*p* ≤ 0.05 and α = 0.01)

Overall mean metAFLP characteristics were compared for the different genotype sets and regeneration methods (ANOVA; see Fig. 4 for *F* statistics). The S^1^ genotype exhibited markedly lower values than the S^2^, S^3^, and S^4^ sets. No statistically significant differences were noted between the S^2^, S^3^, and S^4^ groups for any of the regeneration methods. For the shed microspore culture approach, S^2^ was similar to both S^1^ and S^3^/S^4^ (Fig. 4).

#### Comparison of genotype sets and different regeneration methods with individual metAFLP characteristics

**Fig. 5:**
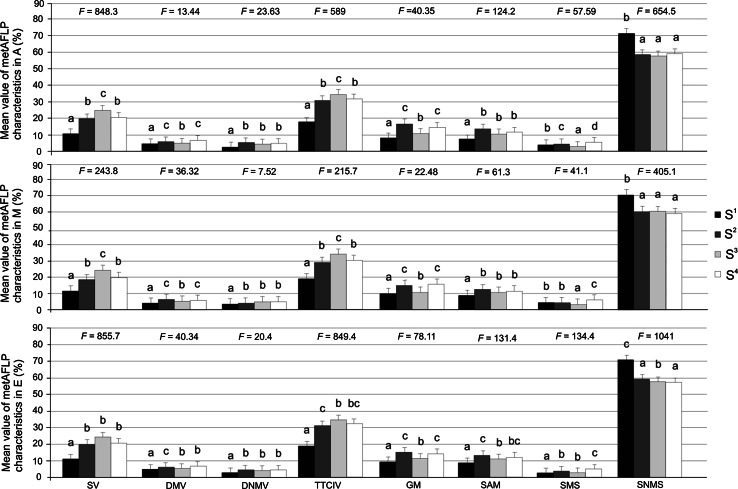
Grouping of genotype sets (Tukey’s multiple range test at 5 % significance level) for individual metAFLP characteristics with respect to plant regeneration method with *error bars*. For each regeneration approach, statistically similar genotype sets (S^i^, where i = 1–4) are indicated with the same letter. *F* indicates ANOVA statistics (*p* ≤ 0.05 and α = 0.01). Abbreviations as in Figs. 3 and 4

Individual metAFLP characteristics were compared for the different genotype sets and regeneration methods (ANOVA; see Fig. 5 for *F* statistics). With the exception of SMS, patterns of similarity between the genotype sets were identical between the shed microspore and anther regeneration methods. For these regeneration approaches, no significant differences were observed between the S^2^ and S^4^ genotypes for SV, DMV, TTCIV, and GM, and there were no significant differences between S^2^, S^3^, and S^4^ for DNMV, SAM, and SNMS. Genomic set groupings were generally different for immature zygotic embryo-derived regeneration compared to the other two regeneration approaches.

#### Comparison of metAFLP characteristics

**Fig. 6:**
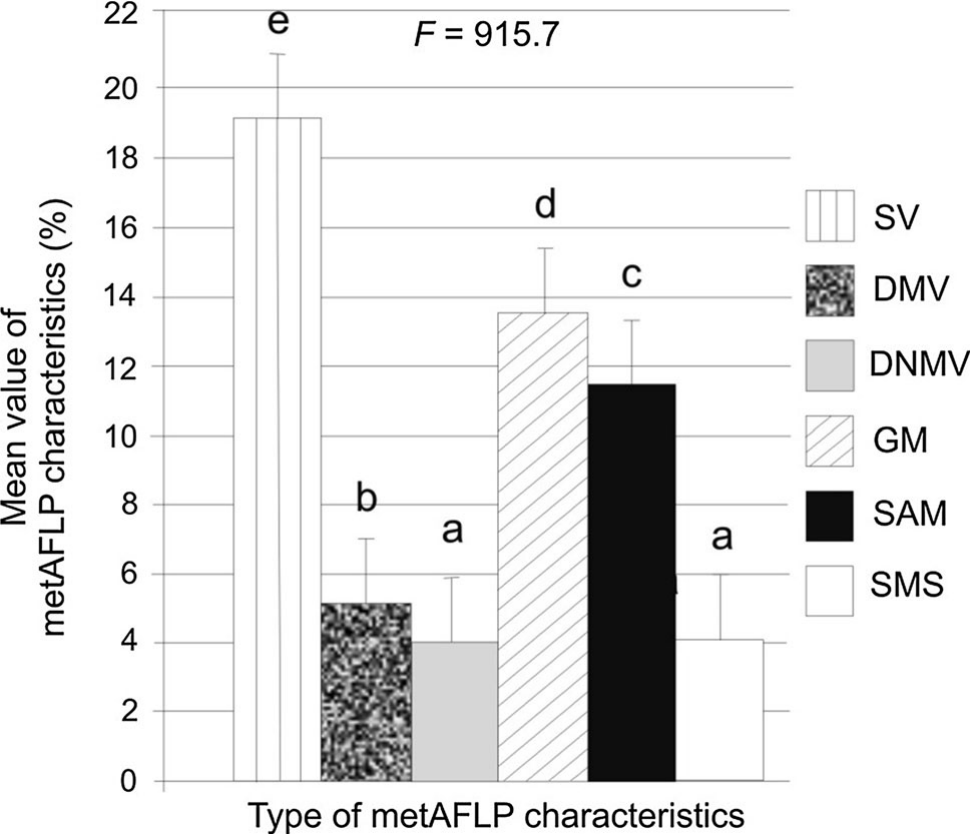
Grouping of metAFLP characteristics (Tukey’s multiple range test at 5 % significance level) with *error bars*. Statistically similar characteristics are indicated by the *same letter*. *F* indicates ANOVA statistics (*p* ≤ 0.05 and α = 0.01). Abbreviations as in Fig. 3

Significant differences were observed between the different metAFLP characteristics with the exception of DNMV and SMS (Tukey’s test, Fig. 6).

Comparison of GM and global DNA methylation data from metAFLP and RP-HPLC analyses.

In our previous study, we observed a decrease in global DNA methylation of regenerants compared to donors irrespective of the in vitro tissue culture approach used for plant regeneration. RP-HPLC showed that global DNA methylation of donors and regenerants was 25.39 and 24.1 %, respectively (Machczyńska et al. 2014b). MetAFLP estimates of GM of the restriction sites in regenerants were twofold lower (Table 2) than RP-HPLC values. RP-HPLC estimated the decrease in global DNA methylation between donors and regenerants to be ~1.29 %. While GM values were lower using metAFLP, the decrease in methylation between donors and regenerants was also estimated as ~1 % (DNMV–DMV; Table 2).

RP-HPLC and metAFLP estimates of global DNA methylation and GM were compared using Pearson correlation analysis. When genotype sets and in vitro tissue culture regeneration approaches were not taken into consideration, limited correlation was observed (*r* = 0.15, *p* < 0.02). When sets were considered separately, a high level of correlation between RP-HPLC and metAFLP was seen for S^1^ but not for S^2^–S^4^ (Table 3).

**Table 3:** Pearson correlation coefficients comparing genome methylation (GM) estimates by metAFLP and global DNA methylation by RP-HPLC in different genotype sets

Set (S^i^)	Pearson correlation coefficient (*r*); *p* value
S^1^	0.38; *p* < 0.001
S^2^	−0.17; *p* < 0.27
S^3^	−0.06; *p* < 0.70
S^4^	0.09; *p* < 0.57

S^i^ represents genotype, where i = 1–4

Pearson correlation was also used to compare estimates of global DNA methylation by RP-HPLC and GM by metAFLP for the different in vitro plant regeneration approaches (Table 4). Significant correlations were observed for the anther- and embryo-derived, but not the shed microspore-derived, regenerants.

**Table 4:** Pearson correlation comparing genome methylation estimates by metAFLP and global DNA methylation estimates by RP-HPLC for different plant regeneration approaches

In vitro tissue culture regeneration approaches	Pearson correlation coefficient (*r*); *p* value
Anther culture	−0.28; *p* < 0.01
Shed microspore culture	0.15; *p* < 0.28
Immature zygotic embryo culture	0.28; *p* < 0.01

## Discussion

Several studies have compared the molecular characteristics of tissue culture-derived regenerants and their donor plants (Bouman and Klerk 2001; Hossain et al. 2003; Bhatia et al. 2009). However, the plants used as a source of explants in these studies were not assessed for uniformity (Teyssier et al. 2013), and this may be of critical importance for the quantitative evaluation of TCIV (Bednarek et al. 2007; Machczyńska et al. 2014a). To address this, we used specific plant resources to examine TCIV in this study. Four randomly selected and genetically distinct DH genotypes derived from isolated microspores from the cv. Bogo triticale served as donor plants. DH donor plants were cloned by partitioning plant clumps after tillering. This gave sufficient numbers of explants to produce simultaneous androgenic and somatic regenerants for quantitative analysis. To ensure that cloned individuals were epigenetically and genetically identical, individuals were analyzed using the same primer combinations as for analysis of genotype sets. A lack of morphological and minor molecular differences between cloned individuals from the same donor plant were seen (0–1.8 % for the *Acc*65I/*Mse*I metAFLP platform and 0–1.2 % for the *Kpn*I/*Mse*I digest), but these could be attributed to the metAFLP experimental error (Meudt and Clarke 2007). Although we did our best to pick leaves for DNA isolation at the same developmental stages, some differences among samples may have not been excluded. The differences observed with the *Acc*65I/*Mse*I digest related to DNA methylation may reflect this. It cannot be excluded that variation between cloned individuals of the same genotype might be a consequence of genomic factors such as activity of mobile elements (Schwartz and Dennis 1986; Khan et al. 2013) or of DNA polymerase slippage (Alhani and Wilkinson 1998). Nevertheless, a sufficient number of cloned individuals were identified that had no detectable variation using the *Acc*65I/*Mse*I and *Kpn*I/*Mse*I metAFLP platforms. Of 53 clones, 39 individuals representing four genotypes of cv. Bogo were used for plant regeneration via the A, M, and E approaches.

Recently, we described an extended metAFLP approach that facilitated the evaluation of numerous quantitative characteristics (Machczyńska et al. 2014a). The observed TCIV was higher than that found in barley (Bednarek et al. 2007) and, whereas methylation pattern changes were predominant in barley, most of the observed variation in preliminary triticale study was a result of sequence changes (Machczyńska et al. 2014a). The level of SV observed in the regenerants could be attributed to the activation of transposable elements that is thought to take place in parallel with genomic DNA demethylation (Brettell and Dennis 1991; Liu et al. 2004) in response to abiotic stresses (Kashkush et al. 2003). The number of SAM was comparable to the level of sequence alterations. This suggested that tissue culture prompted numerous sequence and DNA methylation changes in triticale, possibly reflecting previously observed genome instabilities (Lapitan et al. 1984; Bento et al. 2011) such as chromosome rearrangements (Oleszczuk et al. 2011). The epigenetic and genetic changes did not cause any readily apparent morphological consequences in the regenerants, suggesting that the changes may have occurred in genomic regions that did not affect morphological traits. Alternatively, changes in DNA methylation pattern and/or sequence mutations affecting essential traits might be rare, or regenerants with such changes might be eliminated during plant regeneration. It is possible that the number of regenerants analyzed may have been sufficient to detect epigenetic and genetic variation, but not to identify morphological mutants. However, changes in DNA methylation level caused phenotypic variation in other organisms, such as *Linaria* flower (Cubas et al. 1999) and *Arabidopsis thaliana* (Soppe et al. 2000), and the DNA methylation-derived changes were heritable (Kathiria et al. 2010). Thus, even though no phenotypic variation was observed in triticale regenerants, successive regenerant progenies may display morphological changes.

It is possible that donor plant genotype may influence TCIV. Our evaluation of four sample sets comprising distinct donor genotypes allowed examination of the role of genotype on TCIV. We previously demonstrated that donor genotype had an effect on TCIV in barley (Bednarek et al. 2007). However, in that case, it was possible that the observed “donor effect” was a statistical artifact of the Tukey–Kramer test which, when used to compare variable numbers of regenerants within a data set, compares sets in pairs and is capable of delivering a non-existent “genotype effect”. The genotype sets in the present study also contained different numbers of regenerants. The most different set was represented by the largest number of regenerants, which may favor the non-existent “genotype effect” explanation. The S^1^ set contained highly uniform regenerants, which resulted in lower metAFLP values compared to the other sets. Tukey’s tests for metAFLP characteristics distinguished between most of the sets, which favored the donor effect hypothesis.

Another factor that may contribute to TCIV is the method used for in vitro plant regeneration (Bairu et al. 2011). In triticale, DHs may be produced by androgenesis (Immonen and Robinson 2000; Oleszczuk et al. 2004; Würschum et al. 2012) or by artificial crossing with maize (Wȩdzony et al. 1998), wheat (Pratap et al. 2005), or pearl millet (Inagaki and Hash 1998). Artificial crossing necessitates the use of chromosome doubling agents that may generate additional variation (Liu et al. 2009; Wu et al. 2012; Wang et al. 2014). In androgenesis, spontaneous doubling rate in triticale varies from 0 to 50 % (Arzani and Darvey 2001; Ślusarkiewicz-Jarzina and Ponitka 2003; Oleszczuk et al. 2004; Lantos et al. 2014). Androgenesis is the most frequently adopted method for the evaluation of DH plants in cereals (Maluszyński et al. 2003) and was therefore used here. By contrast, with isolated microspore (Oleszczuk et al. 2004) or chromosome elimination methods (Powell et al. 1986), plant regeneration using anthers or immature zygotic embryos as sources of tissues can proceed via an intermediate callus stage (Kim et al. 2003; Seguí-Simarro and Nuez 2007). Numerous previous studies (Phillips et al. 1994; Young et al. 1999; Wang et al. 2013) suggested that the presence of the callus stage was the most probable source of TCIV. In our experiments, E and A regeneration involved an intermediate callus phase; however, no significant differences in TCIV were observed between regeneration approaches when genotype sets were analyzed together. These results were consistent with those for barley (Bednarek et al. 2007), where the levels of TCIV in androgenesis and immature zygotic embryo-derived regenerants were comparable. This is also consistent with studies using *Freesia hybrida*, in which sequence and methylation changes were similar for direct regeneration and indirect regeneration proceeding via the callus stage (Gao et al. 2010). By contrast, studies in barley revealed a higher level of DNA methylation in regenerants from anther culture than in those derived from *Hordeum bulbosum* (Devaux et al. 1993). When tissue culture methods were compared for each individual genotype in our dataset, differences in TCIV between regeneration approaches were observed. Observed differences were mostly attributable to the S^1^ set, which supported either the donor hypothesis or the statistically-derived “genotype effect”. However, individual metAFLP characteristics were more discriminative: some metAFLP types distinguished most of the sets, while others separated S^1^ only. Thus, it is possible that plant regeneration approaches influence TCIV but that the differences are a result of subtle effects (possibly random fluctuations) that can only be observed by comparison of different metAFLP characteristics.

Assuming that metAFLP can quantify GM through examination of the restriction sites recognized by the isoschizomers used in the approach and that RP-HPLC can be used to evaluate global DNA methylation, it might be expected that outcomes of the two methods would be correlated; however, the expected correlation was not apparent. MetAFLP uses changes at restriction sites recognized by *Acc*65I and *Kpn*I isoschizomers as a proxy for whole-GM; however, it is possible that this may not reflect changes affecting the whole genome accurately. This suggests that the restriction sites used in metAFLP might not be randomly distributed across chromosomes. Genetic mapping with AFLP markers in rye showed that mapped markers were not evenly distributed across chromosomes but formed clusters of tightly linked markers (Bednarek et al. 2003). If this is the case with the restriction sites used here, then the weak correlation between the metAFLP and RP-HPLC estimates of GM and global DNA methylation suggests that metAFLP reflects differences within particular genomic regions rather than across the whole genome.

It was previously demonstrated that global DNA methylation, as estimated using RP-HPLC analysis, decreased in regenerants compared to donors irrespective of the regeneration approaches used (Machczyńska et al. 2014b). The decrease in DNA methylation was about 1.2 %, which is consistent with comparative methylation values from the metAFLP approach. However, RP-HPLC and metAFLP global DNA methylation and GM were poorly correlated for all genotype sets except S^1^, which exhibited relatively high correlation. The correlation for the S^1^ set may reflect either the larger number of individuals used for the analysis or the putative genotypic effects of the D^1^ genotype. Similar analyses of larger sets would be needed to discriminate between the two hypotheses.

The direction of DNA methylation alteration in triticale differed from that in barley (Bednarek et al. 2007). Presently, it is unclear whether these opposite directions are species- or even cultivar-specific. One may speculate, however, that, as a result of its more recent development and polyploidization, triticale or some of its forms might have a less stable genome than barley (Bento et al. 2008) and might therefore be more prone to genomic changes. However, it remains possible that the opposing directions of change may be attributable to the different genotypes. Cytological evidence suggests that some triticale forms are highly stable under tissue culture treatment, while others exhibit more instability (Oleszczuk et al. 2011). Additional complexity may underlie GM, as demonstrated by the different change directions observed in a single species, barley, using the methylation-sensitive amplified polymorphism (Li et al. 2007) and metAFLP (Bednarek et al. 2007) approaches. Whether these differences reflect genuine genotype effects or simply the differences in the genomic regions examined by the different methodological approaches remains to be investigated.

The metAFLP characteristics used for the evaluation of differences between genotype sets proved to discriminate sets and tissue culture approaches. The characteristics were also distinct from each other. This was consistent with data from barley (Bednarek et al. 2007) and suggested that the different metAFLP quantitative characteristics reflected different phenomena (sequence changes, alterations in site methylation patterns, etc.). If the different metAFP characteristics were not linked to biological phenomena, then they would most likely not have been statistically discriminated from one another. Taken together, our results demonstrate the value of the metAFLP approach for the examination of TCIV and show that such variation is linked to genetic background related to in vitro plant regeneration approaches.

## Conclusions

Regeneration of triticale plants via in vitro tissue culture was error-prone and affected DNA sequence and methylation patterns, irrespective of the culture method used. One of the most frequently observed variation types was the alteration in sequence between donor and regenerant plants. Numerous changes to DNA methylation pattern were also observed in regenerants compared to donors. Observed changes initially appeared unlinked to donor genotype or the tissue regeneration approach used; however, differences became apparent when individual metAFLP characteristics were examined. Both metAFLP and RP-HPLC estimates of GM and global DNA methylation indicated a decrease in methylation in regenerants compared to donors; however, RP-HPLC methylation estimates were in most cases poorly correlated with estimates of methylation from metAFLP examination of isoschizomer restriction sites. In summary, metAFLP quantitative characteristics were useful for evaluation of TCIV, which appeared to be linked to genotype background and in vitro regeneration approaches.

## Electronic supplementary material

Supplementary material 1 (DOCX 16 kb)

Supplementary material 2 (DOCX 22 kb)

Supplementary material 3 (DOCX 18 kb)
