# Supplementary material for: Tissue culture-induced genetic and epigenetic variation in triticale (× Triticosecale spp. Wittmack ex A. Camus 1927) regenerants
Source: Plant Mol Biol. 2015 Sep 3;89(3):279–92. doi: 10.1007/s11103-015-0368-0 (PMC4579263; doi:10.1007/s11103-015-0368-0)
Supplement: Supplementary file 1 — Supplementary material 1 (DOCX 16 kb) [file 11103_2015_368_MOESM1_ESM.docx]

**Tissue culture-induced genetic and epigenetic variation in triticale (x *Triticosecale* spp. Wittmack ex A. Camus 1927) regenerants**

**Plant Molecular Biology**

Joanna Machczyńska^1*^, Janusz Zimny^2^, Piotr Tomasz Bednarek*^1^

^1^Department of Plant Physiology and Biochemistry

^2^Department of Plant Biotechnology and Cytogenetics

Plant Breeding and Acclimatization Institute-National Research Institute, 05-870 Błonie, Radzików, Poland

^*^Corresponding author: Piotr Tomasz Bednarek; e-mail: [p.bednarek@ihar.edu.pl](mailto:p.bednarek@ihar.edu.pl); phone number: +48 22 7334535; fax number: +48 22 7254714

**Online Resource 1** Oligonucleotides exploited for metAFLP in studies on triticale cv. Bogo

| **MetAFLP oligomer** | **Sequence 5’→ 3’** |
| --- | --- |
| Adapter1 Acc65I | CTCGTAGCATGCGTACA |
| Adapter2 Acc65I | GTACTGTACGCATGCTAC |
| Adapter1 KpnI | CTCGTAGCATGCGTACAGTAC |
| Adapter2 KpnI | TGTACGCATGCTAC |
| Adapter1 Mse | TACTCAGGACTCATC |
| Adapter2 Mse | GAGTCCTGAGTAGCAG |
| **Preselective primers** |  |
| Acc65I/KpnI | GCATGCGTACAGTACC |
| MseI | GATGAGTCCTGAGTAAC |
| **Labeled ^32^P Acc65I/KpnI selective oligonucleotides** |  |
| CpG GCA | CATGCGTACAGTACCGCA |
| CpG GGC | CATGCGTACAGTACCGGC |
| CpG GAC | CATGCGTACAGTACCGAC |
| CpG ACG | CATGCGTACAGTACCACG |
| CpG TCG | CATGCGTACAGTACCTCG |
| CpXpG ATG | CATGCGTACAGTACCATG |
| CpXpG (A/T)GG | CATGCGTACAGTACC(A/T)GG |
| CpXpG AGG | CATGCGTACAGTACCAGG |
| CpXpG AGA | CATGCGTACAGTACCAGA |
| CpXpG AGC | CATGCGTACAGTACCAGC |
| CpXpG TGC | CATGCGTACAGTACCTGC |
| CpXpG TTG | CATGCGTACAGTACCTTG |
| CpXpX ATT | CATGCGTACAGTACCATT |
| CpXpX TAA | CATGCGTACAGTACCTAA |
| **MseI selective oligonucleotides** |  |
| M CTC | GATGAGTCCTGAGTAACTC |
| M CTG | GATGAGTCCTGAGTAACTG |
| M CTA | GATGAGTCCTGAGTAACTA |
| M CTT | GATGAGTCCTGAGTAACTT |
| M CAC | GATGAGTCCTGAGTAACAC |
| M CAG | GATGAGTCCTGAGTAACAG |
| M CAT | GATGAGTCCTGAGTAACAT |
| M CAA | GATGAGTCCTGAGTAACAA |
| M CGC | GATGAGTCCTGAGTAACGC |
| M CGT | GATGAGTCCTGAGTAACGT |
| M CCG | GATGAGTCCTGAGTAACCG |
| M CCT | GATGAGTCCTGAGTAACCT |
