# Supplementary material for: Tissue culture-induced genetic and epigenetic variation in triticale (× Triticosecale spp. Wittmack ex A. Camus 1927) regenerants
Source: Plant Mol Biol. 2015 Sep 3;89(3):279–92. doi: 10.1007/s11103-015-0368-0 (PMC4579263; doi:10.1007/s11103-015-0368-0)
Supplement: Supplementary file 2 — Supplementary material 2 (DOCX 22 kb) [file 11103_2015_368_MOESM2_ESM.docx]

**Tissue culture-induced genetic and epigenetic variation in triticale (x *Triticosecale* spp. Wittmack ex A. Camus 1927) regenerants**

**Plant Molecular Biology**

Joanna Machczyńska^1^, Janusz Zimny^2^, Piotr Tomasz Bednarek*^1^

^1^Department of Plant Physiology and Biochemistry

^2^Department of Plant Biotechnology and Cytogenetics

Plant Breeding and Acclimatization Institute-National Research Institute, 05-870 Błonie, Radzików, Poland

^*^Corresponding author: Piotr Tomasz Bednarek; e-mail: [p.bednarek@ihar.edu.pl](mailto:p.bednarek@ihar.edu.pl); phone number: +48 22 7334535; fax number: +48 22 7254714

**Online Resource 2** The number of amplified fragments in adequate primer combinations. TNAF – total number of amplified fragments, PFA – polymorphic fragments in *Acc*65I/*Mse*I, PFK – polymorphic fragments in *Kpn*I/*Mse*I, MFA – monomorphic fragments in *Acc*65I/*Mse*I, MFK – monomorphic fragments in *Kpn*I/*Mse*I, PFAK – common polymorphic fragments for *Acc*65I and *Kpn*I, MFAK – common monomorphic fragments for *Acc*65I and *Kpn*I. S^1^, S^2^, S^3^, S^4^ – sets of plant material derived from four different genotypes of triticale cv. Bogo

| Selective primer combination | Set  material | TNAF | PFA | PFK | MFA | MFK | PFAK | MFAK |
| --- | --- | --- | --- | --- | --- | --- | --- | --- |
| CpG GCA/M CGC | S^1^ | 28 | 11 | 6 | 17 | 22 | 4 | 15 |
|  | S^2^ | 38 | 23 | 22 | 15 | 16 | 25 | 2 |
|  | S^3^ | 41 | 28 | 17 | 13 | 24 | 14 | 10 |
|  | S^4^ | 31 | 15 | 10 | 16 | 21 | 8 | 14 |
| CpG GGC/M CTG | S^1^ | 32 | 21 | 22 | 11 | 10 | 17 | 6 |
|  | S^2^ | 42 | 30 | 22 | 12 | 20 | 25 | 2 |
|  | S^3^ | 62 | 35 | 25 | 27 | 37 | 28 | 17 |
|  | S^4^ | 51 | 30 | 19 | 21 | 32 | 22 | 11 |
| CpG GAC/M CAA | S^1^ | 42 | 25 | 21 | 17 | 21 | 16 | 12 |
|  | S^2^ | 62 | 40 | 17 | 22 | 45 | 17 | 15 |
|  | S^3^ | 63 | 32 | 23 | 31 | 40 | 24 | 22 |
|  | S^4^ | 54 | 33 | 17 | 21 | 37 | 21 | 10 |
| CpG ACG/M CAT | S^1^ | 40 | 12 | 7 | 28 | 33 | 8 | 25 |
|  | S^2^ | 36 | 12 | 8 | 24 | 28 | 10 | 20 |
|  | S^3^ | 56 | 13 | 14 | 43 | 42 | 14 | 35 |
|  | S^4^ | 42 | 12 | 7 | 30 | 35 | 10 | 20 |
| CpG TCG/M CGT | S^1^ | 47 | 34 | 29 | 13 | 18 | 28 | 10 |
|  | S^2^ | 43 | 17 | 20 | 26 | 23 | 18 | 16 |
|  | S^3^ | 64 | 33 | 37 | 31 | 27 | 30 | 22 |
|  | S^4^ | 48 | 29 | 27 | 19 | 21 | 25 | 15 |
| CpXpG ATG/M CTC | S^1^ | 43 | 15 | 15 | 28 | 28 | 11 | 24 |
|  | S^2^ | 47 | 26 | 21 | 21 | 22 | 21 | 19 |
|  | S^3^ | 48 | 20 | 15 | 28 | 33 | 12 | 24 |
|  | S^4^ | 40 | 19 | 20 | 21 | 20 | 16 | 14 |
| CpXpG (A/T)GG/M CCT | S^1^ | 48 | 16 | 11 | 32 | 37 | 9 | 30 |
|  | S^2^ | 38 | 17 | 15 | 21 | 23 | 12 | 13 |
|  | S^3^ | 56 | 20 | 15 | 36 | 41 | 15 | 33 |
|  | S^4^ | 52 | 25 | 14 | 27 | 38 | 13 | 20 |
| CpXpG AGG/M CAG | S^1^ | 33 | 15 | 18 | 18 | 15 | 13 | 13 |
|  | S^2^ | 47 | 27 | 23 | 20 | 24 | 23 | 14 |
|  | S^3^ | 67 | 36 | 35 | 31 | 32 | 34 | 19 |
|  | S^4^ | 48 | 29 | 22 | 19 | 26 | 20 | 15 |
| CpXpG AGA/M CAA | S^1^ | 51 | 29 | 26 | 22 | 25 | 19 | 15 |
|  | S^2^ | 58 | 32 | 23 | 26 | 35 | 22 | 12 |
|  | S^3^ | 64 | 36 | 26 | 28 | 38 | 28 | 23 |
|  | S^4^ | 64 | 33 | 11 | 31 | 53 | 13 | 25 |
| CpXpG AGC/M CTA | S^1^ | 37 | 18 | 13 | 19 | 24 | 12 | 18 |
|  | S^2^ | 41 | 19 | 19 | 22 | 22 | 16 | 10 |
|  | S^3^ | 44 | 17 | 18 | 27 | 26 | 16 | 23 |
|  | S^4^ | 66 | 35 | 27 | 31 | 39 | 20 | 12 |
| CpXpG TGC/M CCG | S^1^ | 43 | 28 | 24 | 15 | 19 | 22 | 13 |
|  | S^2^ | 38 | 25 | 15 | 13 | 23 | 14 | 9 |
|  | S^3^ | 61 | 37 | 33 | 24 | 28 | 30 | 21 |
|  | S^4^ | 42 | 24 | 20 | 18 | 22 | 20 | 16 |
| CpXpG TTG/M CTT | S^1^ | 47 | 18 | 22 | 29 | 25 | 18 | 25 |
|  | S^2^ | 46 | 18 | 22 | 28 | 24 | 15 | 17 |
|  | S^3^ | 51 | 20 | 22 | 31 | 29 | 17 | 21 |
|  | S^4^ | 62 | 29 | 18 | 33 | 44 | 18 | 26 |
| CpXpX ATT/M CAC | S^1^ | 49 | 33 | 34 | 16 | 15 | 26 | 8 |
|  | S^2^ | 54 | 29 | 29 | 25 | 25 | 25 | 19 |
|  | S^3^ | 57 | 26 | 29 | 31 | 28 | 24 | 23 |
|  | S^4^ | 62 | 39 | 26 | 23 | 36 | 25 | 15 |
| CpXpX TAA/M CGT | S^1^ | 44 | 35 | 30 | 9 | 14 | 28 | 6 |
|  | S^2^ | 53 | 35 | 32 | 18 | 21 | 28 | 20 |
|  | S^3^ | 49 | 28 | 27 | 21 | 22 | 23 | 12 |
|  | S^4^ | 48 | 36 | 32 | 12 | 16 | 30 | 9 |
| Total |  | 2720 | 1429 | 1172 | 1291 | 1544 | 1072 | 935 |
