# Supplementary material for: Tissue culture-induced genetic and epigenetic variation in triticale (× Triticosecale spp. Wittmack ex A. Camus 1927) regenerants
Source: Plant Mol Biol. 2015 Sep 3;89(3):279–92. doi: 10.1007/s11103-015-0368-0 (PMC4579263; doi:10.1007/s11103-015-0368-0)
Supplement: Supplementary file 3 — Supplementary material 3 (DOCX 18 kb) [file 11103_2015_368_MOESM3_ESM.docx]

**Tissue culture-induced genetic and epigenetic variation in triticale (x *Triticosecale* spp. Wittmack ex A. Camus 1927) regenerants**

**Plant Molecular Biology**

Joanna Machczyńska^1^, Janusz Zimny^2^, Piotr Tomasz Bednarek*^1^

^1^Department of Plant Physiology and Biochemistry

^2^Department of Plant Biotechnology and Cytogenetics

Plant Breeding and Acclimatization Institute-National Research Institute, 05-870 Błonie, Radzików, Poland

^*^Corresponding author: Piotr Tomasz Bednarek; e-mail: [p.bednarek@ihar.edu.pl](mailto:p.bednarek@ihar.edu.pl); phone number: +48 22 7334535; fax number: +48 22 7254714

**Online Resource 3** Arrangement of the number of 4 – digit binary codes calculated for each set of plant material with and without considering *in vitro* tissue culture regeneration approaches. The S^1^, S^2^, S^3^ and S^4^ abbreviations stand for the sets derived from four different genotypes of triticale cv. Bogo. The R_A_, R_M_, R_E_ reflect regenerants derived from anther, shed microspore and immature zygotic embryo cultures, respectively

| metAFLP profile | S^1^ | | | S^2^ | | | S^3^ | | | S^4^ | | |
| --- | --- | --- | --- | --- | --- | --- | --- | --- | --- | --- | --- | --- |
|  | R_A_ | R_M_ | R_E_ | R_A_ | R_M_ | R_E_ | R_A_ | R_M_ | R_E_ | R_A_ | R_M_ | R_E_ |
| 0000 | 1046 | 1225 | 1106 | 929 | 213 | 610 | 602 | 242 | 524 | 466 | 454 | 615 |
| 0001 | 182 | 204 | 165 | 237 | 59 | 145 | 154 | 62 | 123 | 63 | 65 | 61 |
| 0010 | 266 | 344 | 316 | 478 | 90 | 310 | 360 | 134 | 216 | 326 | 286 | 453 |
| 0011 | 429 | 563 | 349 | 499 | 111 | 307 | 334 | 145 | 266 | 452 | 442 | 552 |
| 0100 | 119 | 199 | 73 | 135 | 32 | 130 | 157 | 45 | 129 | 119 | 75 | 127 |
| 0101 | 228 | 262 | 294 | 217 | 41 | 150 | 327 | 147 | 154 | 145 | 138 | 234 |
| 0110 | 66 | 92 | 31 | 95 | 22 | 56 | 95 | 28 | 83 | 49 | 33 | 75 |
| 0111 | 539 | 561 | 656 | 666 | 172 | 512 | 651 | 269 | 515 | 447 | 415 | 586 |
| 1000 | 192 | 220 | 238 | 469 | 88 | 306 | 617 | 267 | 413 | 296 | 262 | 384 |
| 1001 | 43 | 83 | 50 | 96 | 25 | 55 | 164 | 66 | 91 | 78 | 48 | 78 |
| 1010 | 151 | 238 | 167 | 628 | 135 | 389 | 1546 | 598 | 843 | 513 | 494 | 705 |
| 1011 | 339 | 491 | 378 | 639 | 101 | 368 | 513 | 215 | 362 | 429 | 350 | 431 |
| 1100 | 168 | 217 | 117 | 180 | 50 | 118 | 170 | 56 | 212 | 113 | 103 | 155 |
| 1101 | 272 | 290 | 297 | 641 | 152 | 466 | 589 | 227 | 439 | 397 | 403 | 539 |
| 1110 | 275 | 350 | 298 | 254 | 49 | 246 | 449 | 168 | 285 | 141 | 113 | 183 |
| 1111 | 10285 | 12181 | 10649 | 7939 | 1865 | 5447 | 8932 | 3595 | 7090 | 5196 | 4839 | 6892 |
